# Design by Nature: Emerging Applications of Native Liver Extracellular Matrix for Cholangiocyte Organoid-Based Regenerative Medicine

**DOI:** 10.3390/bioengineering9030110

**Published:** 2022-03-07

**Authors:** Jorke Willemse, Luc J. W. van der Laan, Jeroen de Jonge, Monique M. A. Verstegen

**Affiliations:** Department of Surgery, Erasmus MC Transplant Institute, University Medical Center, 3015 CN Rotterdam, The Netherlands; j.willemse@erasmusmc.nl (J.W.); l.vanderlaan@erasmusmc.nl (L.J.W.v.d.L.); j.dejonge.1@erasmusmc.nl (J.d.J.)

**Keywords:** extracellular matrix, cholangiocyte organoids, bile duct, liver, tissue engineering, regenerative medicine, culture substrates

## Abstract

Organoid technology holds great promise for regenerative medicine. Recent studies show feasibility for bile duct tissue repair in humans by successfully transplanting cholangiocyte organoids in liver grafts during perfusion. Large-scale expansion of cholangiocytes is essential for extending these regenerative medicine applications. Human cholangiocyte organoids have a high and stable proliferation capacity, making them an attractive source of cholangiocytes. Commercially available basement membrane extract (BME) is used to expand the organoids. BME allows the cells to self-organize into 3D structures and stimulates cell proliferation. However, the use of BME is limiting the clinical applications of the organoids. There is a need for alternative tissue-specific and clinically relevant culture substrates capable of supporting organoid proliferation. Hydrogels prepared from decellularized and solubilized native livers are an attractive alternative for BME. These hydrogels can be used for the culture and expansion of cholangiocyte organoids in a clinically relevant manner. Moreover, the liver-derived hydrogels retain tissue-specific aspects of the extracellular microenvironment. They are composed of a complex mixture of bioactive and biodegradable extracellular matrix (ECM) components and can support the growth of various hepatobiliary cells. In this review, we provide an overview of the clinical potential of native liver ECM-based hydrogels for applications with human cholangiocyte organoids. We discuss the current limitations of BME for the clinical applications of organoids and how native ECM hydrogels can potentially overcome these problems in an effort to unlock the full regenerative clinical potential of the organoids.

## 1. Introduction

The shortage of donor organs is a central theme in the field of liver transplantation, which still is the only curative treatment option for patients suffering from end-stage liver failure. The donor shortage leads to high waiting list mortality. However, suitable donor livers often do not become available in time for up to 20% of the patients on the waiting list [1,2,3]. Efforts made to increase the pool of available donor livers include the use of extended criteria donor organs, such as the use of donation after circulatory death (DCD) organs [4]. The use of DCD livers is associated with a higher incidence of ischemia-type biliary lesions (16% vs. 3% when compared to donation after brain death) [5,6]. Cholangiocytes form an active barrier between the cytotoxic bile and surrounding tissue [7]. They are sensitive to ischemia, and the extra period of warm ischemia in DCD transplantation can cause deficits in the biliary epithelium, such as non-anastomotic strictures [4,8,9]. Ultimately, 65% of patients with ischemic cholangiopathy require retransplantation, as there is currently no alternative treatment option available [6]. This does not only have far-reaching impact on patients, but also reduces the number of available donor grafts for other patients.

Regenerative medicine strategies could repair the biliary deficits while the liver is preserved ex vivo on an organ perfusion setup. Eshmuminov et al. recently showed that it is feasible to maintain human livers on the pump for up to 7 days [10]. These improvements in the field of organ preservation could open up a window of opportunity for ex vivo organ repair. Stem cells or cholangiocytes can be used to repair deficits in the biliary epithelium [11,12].

The aim of this review is to discuss the potential of native human liver extracellular matrix (ECM) for the clinical grade applications of cholangiocyte organoids for regenerative medicine in patients. We discuss why the use of mouse tumor-derived basement membrane extract (BME) is currently limiting the clinical applications of cholangiocyte organoids. In addition, we provide an overview of requirements for clinically relevant culture substrates. We will discuss how liver ECM hydrogels provide an alternative culture substrate and why the use of decellularized liver tissue can unlock the full clinical potential of cholangiocyte organoids.

## 2. The Potential of Organoids in Tissue Regeneration

The in vitro isolation and large-scale expansion of primary cholangiocytes cultured on traditional cell culture plastic is challenging [13]. Alternative sources of cholangiocytes are therefore required. Pluripotent stem cells (embryonic or induced) can be directed towards cholangiocytes, but this requires extensive differentiation of cells [14,15,16]. Moreover, there is the potential risk of aberrant (de)differentiation and teratoma formation [17,18,19]. The use of adult tissue-specific progenitors to grow cholangiocyte organoids, enables cell expansion from relatively small (0.5–1.0 cm^3^) human liver biopsies. These organoids maintain a cholangiocyte-like phenotype in vitro [20].

The first cholangiocyte organoids derived from human liver biopsies were described by Huch et al. in 2015 [21]. This research was based upon the discovery of Leucine-rich repeat-containing G-protein coupled receptor 5 (LGR5) positive stem cells found in the intestine [22], which gave rise to intestinal organoids [23]. A similar approach was described for LGR5+ mouse liver-derived organoids [24], followed by human liver organoid cultures. Huch et al. showed that EPCAM positive cells in human liver biopsies can give rise to spheroid-like cultures with an efficiency of 28% (SD: ±3%) [21]. These liver-derived cholangiocyte organoids have a cholangiocyte-like phenotype and express cholangiocyte markers, such as KRT-7/19 and EPCAM, as well as progenitor markers (e.g., SOX-9, LGR-5). Moreover, they have a high and stable proliferation capacity. Relatively small tissue biopsies can yield clinically relevant numbers of cells in a relatively short time span [25,26]. Moreover, the liver-derived organoids can express and upregulate hepatocyte markers, such as albumin, HNF-4α and CYP-3A4, upon differentiation towards hepatocyte-like cells [21,26,27]. This makes them a potential source of both cholangiocytes and hepatocytes. However, the expression of hepatocyte markers and hepatocyte functionality does not yet reach similar levels as primary human hepatocytes.

**Figure 1:**
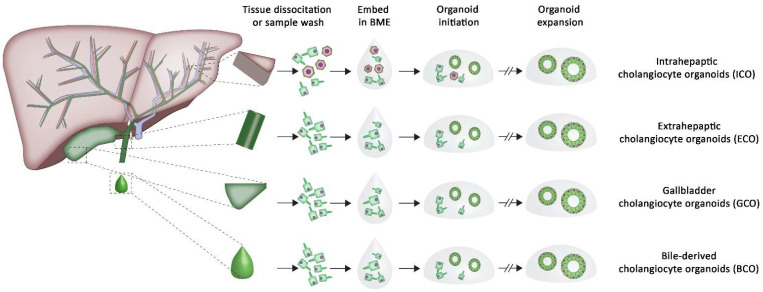
Schematic overview of the workup of cholangiocyte organoids in BME cultures and the different sources of organoids. Cholangiocyte organoids can be initiated from liver tissue biopsies (intrahepatic cholangiocyte organoids; ICO), extrahepatic bile duct tissue biopsies (extrahepatic cholangiocyte organoids; ECO) and gallbladder tissue biopsies (gallbladder cholangiocyte organoids; GCO). Organoids can also be initiated from bile samples (bile-derived cholangiocyte organoids; BCO). Organoids are traditionally grown and expanded in BME.

With the expansion of hepato-pancreato and biliary organoid research, a consistent nomenclature was proposed, and the liver-derived organoids were renamed to intrahepatic cholangiocyte organoids (ICO) to better reflect their origin [20] (see Figure 1 for an overview of the different cholangiocyte organoids and sources). Cholangiocyte organoids were established from gallbladder tissue (gallbladder cholangiocyte organoids; GCO) [28], and from the cholangiocytes’ inner lining the extrahepatic bile duct (EBD, extrahepatic cholangiocyte organoids; ECO) [27,29]. Organoids were also initiated from the circulating EPCAM positive cells in fresh bile samples (bile-derived cholangiocyte organoids, BCO) [30]. These organoids all share similar phenotypic features when cultured, as they all grow in similar spherical structures with comparable proliferation rates [27,30,31]. They express similar progenitor and mature cholangiocyte markers, but are uniquely related to their original tissue of origin and show subtle differences. Rimland et al. showed that differences related to the regional origin of the organoids were retained between ICO, ECO and GCO [31]. In addition, only ICO have the potential to upregulate hepatocyte markers when grown in hepatocyte differentiation conditions [27].

## 3. Repairing Damaged Organs Using Cholangiocyte Organoids

The ability to generate large numbers of cells from relatively small (patient-derived) biopsies is of interest for regenerative cell therapy applications. Therefore, cholangiocyte organoid-derived cells could be a promising source of hepatobiliary cells for organ repair applications [32,33,34]. ICO differentiated towards hepatocyte-like cells were capable of engrafting a damaged mouse liver. These cells showed some level of hepatocyte functionality as human albumin was detected 120 days after engrafting [21]. However, engraftment rates were extremely low (<1%). Therefore, it is not likely that ICO will be used as a cell source for hepatocyte transplantation, as long as current hepatocyte differentiation protocols and cell administration protocols are not improved.

**Figure 2:**
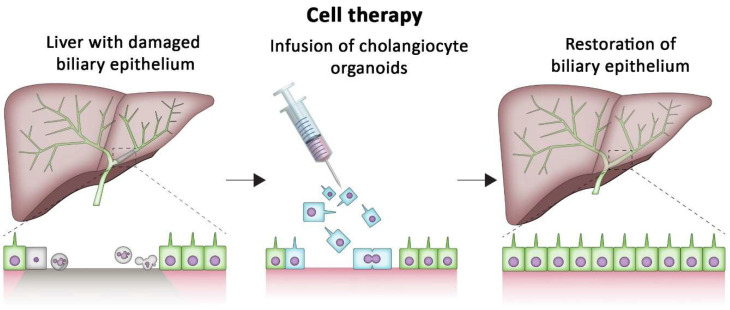
Cholangiocyte organoids can repair deficits in the biliary epithelium. This treatment can restore adequate drainage of bile and prevent build-up of toxic bile inside the liver. Cholangiocyte organoids can be infused into the biliary tree to repair the damaged biliary tree.

Cholangiocyte organoids can also yield cholangiocyte-like cells without the need for additional differentiation steps. Therefore, these cells are of interest for the repair of damaged biliary epithelium. Recently, ground-breaking proof of concept was given by Sampaziotis et al. They showed that cholangiocyte organoids can successfully repair deficits in the biliary epithelium of intrahepatic bile ducts after injecting cells that were derived from the organoids into the biliary tree of a mouse model (Figure 2) [35]. They also showed that GCO can be used to repair deficits of the biliary epithelium of human livers ex vivo while the liver was perfused on a normothermic machine perfusion device. This shows the enormous clinical potential of cholangiocyte organoids.

## 4. Basement Membrane Extract as Culture Substrates

The clinical application of cholangiocyte organoids is currently limited by the use of non-GMP (good manufacturing practice)-compliant basement membrane extracts (BMEs) in which organoids are typically cultured. BME is a complex mixture of extracellular matrix (ECM) components derived from the tumor mass produced by Englebreth-Holm-Swarm (EHS) mouse cells. These cells produce an abundance of basement membrane components, which can be extracted and processed into hydrogels [36,37]. The main constituents of BME are laminin-111, collagen type IV and enactin [37,38,39]. Other components include a myriad of other bioactive ECM components and growth factors [39]. The extraction of these components and subsequent reconstitution was first described in 1986 by Kleinman et al. [36]. They described a protocol where the EHS cells were propagated by transplantation in mice and basement membrane components were extracted from the tumor mass by breaking up protein–protein bonds. The subsequent viscous liquid solidified into a hydrogel at 37 °C and was later commercialized under the name Matrigel [37,38,39,40]. Nowadays, different BME formulations (e.g., growth factor reduced or collagen type IV enriched) are commercially available from various manufacturers (e.g., Corning Matrigel or Cultrex BME). Exact production methods are proprietary information and could therefore differ from previously described protocols.

BME is typically used as an in vitro replacement of the ECM, as it creates a bioactive and biodegradable 3D environment for the cells. Commercially available BMEs are ready-to-use formulations as no additional chemicals are required for solidification of the pre-gel solutions. The non-cytotoxic viscous pre-gel solution remains a liquid at 4 °C and solidifies into a relatively soft hydrogel at 37 °C. Moreover, the optical clarity of the extracts allows for day-to-day monitoring. Therefore, BMEs have long been the golden standard for many different types of in vitro assays, such as angiogenesis assays [41,42], (tumor) cell migration assays [43] or for maintaining (induced) pluripotent stem cells undifferentiated [19,44,45] (see Kleinman et al. [46] and Benton et al. [47] for brief overviews on these applications). BME is used for the expansion of organoids for similar reasons. The bioactive components allow the epithelial cells to self-organize into their typical organoid structures.

However, there are also disadvantages of the use of BME for the culture of organoids. The exact composition is poorly defined and large batch-to-batch differences have been reported [38,44,48]. BME also keeps cells in an undifferentiated and proliferative state [19,39]. This could also hamper the differentiation of cholangiocyte organoids towards mature hepatocytes. Moreover, the constituents are not specific for liver tissue. Under normal circumstances, the main constituent of BME (laminin-111) is not found in healthy adult liver parenchymal regions [49,50]. However, in situations where proliferating cells are required, such as during embryonic development or regeneration after damage, laminin-111 can be found here [49,51,52,53]. Evidence suggests that the presence of laminin can maintain the stemness of certain cells and inhibit the differentiation of hepatic progenitor cells towards mature hepatocytes [52,54,55].

## 5. Tissue-Specific Alternative Culture Substrates

The use of a culture substrate capable of closely mimicking the native liver ECM could drive cells towards mature and functional cholangiocyte and/or improve differentiation of ICO towards hepatocyte-like cells [44,56,57]. Tissue-specific microenvironments can be built using liver ECM components and incorporation of cell signaling moieties (e.g., growth factors). Gradients of growth factors play an important role during embryonic development but are also important for maintaining tissue homeostasis or are involved in tissue repair [53,56,58]. Spatiotemporal deposition of vascular endothelial growth factor (VEGF) through the ECM, for example, guides vascular sprouting during angio(neo)genesis [59,60,61,62]. Growth factors, such as endothelial growth factor (EGF) or hepatocyte growth factor (HGF), are known to maintain the hepatocyte phenotype in cells and are involved in the differentiation of stem cells towards hepatocyte-like cells [63,64]. In addition, biophysical components (e.g., stiffness or elasticity) can influence the behavior of cells through complex mechanotransduction pathways [65,66,67]. This has been shown in vitro with the differentiation of mesenchymal stromal cells, which can be directed by altering matrix elasticity [67,68]. Growing hepatocytes in vitro on rigid matrices decreases their ability to maintain a hepatocyte phenotype [57,69,70,71]. Similarly, an increase in liver ECM stiffness is associated with a reduction in hepatocyte functionality and can lead to liver fibrosis [49,50,51,72]. This shows the importance of having a culture substrate capable of mimicking the native ECM.

Alternative culture substrates have to meet additional requirements. They must, for example, allow for the expansion of the organoids in a GMP-compliant environment. Organoids self-organize into 3D structures through complex cell–cell and cell–matrix interactions. Therefore, the alternative substrate should also allow for the expansion of the organoids by being capable of either deformation or site-specific degradation. Moreover, the growth of cells also implies that the mass-transfer of oxygen, nutrients and metabolic waste products through the substrate is required in order to maintain cell viability while cell numbers are increasing.

### 5.1. Hydrogels as an ECM Mimic

Hydrogels are a promising class of materials for use as in vitro culture substrates [73]. Their polymeric networks are capable of maintaining relatively large amounts of water and can form hydrated environments for cells in vitro. These networks are typically porous and allow for mass-transfer to a certain degree [73]. Hydrogels have found widespread use in biomedical applications such as contact lenses, drug delivery systems and wound dressings [74]. The polymer backbones of hydrogels are typically derived from natural or synthetic sources. Examples of natural polymers used for creating hydrogels are alginate, fibrin, chitosan, cellulose and collagen [73,75,76]. Poly(acrylic acid), poly(vinyl alcohol) and poly(ethylene glycol) (PEG) are examples of synthetic polymers [74,76]. These polymers often require chemical modifications to create cross-linkable and/or degradable networks [73].

Synthetic hydrogels are generally well defined and can be modified with relative ease. This allows for the adjustment of, for example, the number of physical crosslinking sites, which can alter the stiffness of the hydrogel. However, synthetic hydrogels often lack the complexity of the native ECM in terms of mixtures of different bioactive ECM molecules or degradation sites for cells [77]. PEG-based hydrogels were tested for expansion and differentiation of intestinal organoids, but required incorporation of ECM components (e.g., fibronectin or laminin), cell adhesion motifs (RGD) or matrix metalloproteinase-degradable sites [77,78]. Nonetheless, Gjorevski et al. showed that relatively stiff hydrogels (~1.3 kPa) increased the proliferation of these organoids, whereas differentiation to hepatocyte-like cells was more optimal in a softer hydrogel [78]. Similar synthetic hydrogels also support expansion and differentiation of human ICO, but still required the addition of ECM components or cell-adhesion motifs [79]. Ye et al. used a hydrogel based on polyisocyanopeptides (PIC) supplemented with laminin-111 and showed that ICO have similar proliferation rates when compared to ICO grown in BME [80]. Nanocellulose hydrogels appeared to promote differentiation towards hepatocytes [81]. However, it remains elusive whether these hydrogels can fully replace BME, as initiation of ICO in these hydrogels was not tested. Moreover, nanocellulose hydrogels also required supplementation with ECM-components and it was suggested by authors that further optimization regarding addition of ECM-components could enhance ICO growth [81].

In short, creating a tissue-specific microenvironment capable of mimicking the native ECM using synthetic hydrogels requires extensive experience in biochemistry and bioengineering. Fine-tuning an optimal tissue-specific culture substrate is practically not possible without the use of high-throughput screening methods [82]. However, even when using high throughput screening methods, combining and testing multiple ECM components is challenging and time consuming. This problem of recreating tissue mimicking culture substrates can also be circumvented using extracts derived from decellularized healthy tissues [83].

Liver decellularization procedures have been described for livers from different animals, such as rodents [84,85,86,87] and pigs [58,88,89,90,91,92,93,94], but also for whole human livers [94,95,96]. These protocols aim to remove all cellular components from liver tissue while retaining the architecture of the ECM, including the highly tissue-specific spatiotemporal deposition of the ECM components [97,98,99]. Subsequently, the ECM components can be extracted by solubilizing the matrix using the enzyme pepsin in an acidic environment [100,101,102]. The resulting extracts consist of bioactive and biodegradable ECM components, which can form collagen-based hydrogels without the need for complex chemical modifications [103].

### 5.2. ECM-Based Hydrogels

Similar protocols exist for the extraction of collagen from collagen-rich tissues, such as rat-tail tendon, fish scales or skin tissue [104], for biomedical applications (see [103] for a comprehensive review on clinical applications of collagen-based materials). The pepsin cleaves the non-helical telopeptide regions of collagen fibers but does not affect the helical parts of the collagen. Subsequently, the helical parts are released by the enzyme, which can reassemble themselves in long collagen fibers after the pH is normalized to 7.4 [103,105,106]. Similar gelation occurs after pH of the pepsin solubilized ECM is normalized to 7.4. The viscous pre-gel solution self assembles into a collagen-based hydrogel, which contain numerous ECM components [83,107]. The presence of ECM molecules influence the gelation kinetics (e.g., crosslinking of fibril formation) of the ECM hydrogels [108] and creates complex hydrogels with varying mechanical characteristics.

ECM based hydrogels are derived from biological sources and can, therefore, be subject to biological variations. This can be mitigated by creating large batches of ECM extracts from decellularized tissues. ECM extracts are also less well suited for studying the effects of certain biochemical or biophysical components. The hydrogels are collagen-based and altering the concentration also alters the biophysical characteristics and vice versa. This could be resolved by incorporating the ECM extracts into a synthetic hydrogel. This hybrid hydrogel could allow for alterations in biophysical characteristics (e.g., stiffness) without altering the presence of biological components. Skardal et al. used solubilized liver ECM to decorate simplistic PEG hydrogels [109].

### 5.3. Applications of Liver ECM Extracts

Liver ECM-derived extracts have been investigated for improving in vitro hepatocyte cultures by using them as a supplement for culture medium [110], creating 2D coatings [107,111,112] of cell culture plastics or by creating bioactive 3D environments for hepatocytes [71,100,107,111,112,113,114]. Further applications of liver ECM extracts include the culture of other liver cells, such as hepatic stellate cells or liver sinusoidal endothelial cells [88,115], or to improve the differentiation capacity of adipose derived stromal cells [107] or pluripotent stem cells towards hepatocytes [116,117,118]. The liver ECM extracts are also an attractive alternative culture substrate for the culture of cholangiocyte organoids. Giobbe et al. showed that different endodermal organoids (including ICO) can be cultured in non-tissue-specific ECM-derived hydrogels derived from decellularized porcine small intestinal submucosa (SIS) [119]. SIS mainly consist mostly of a mesh of collagen fibers and contains few other proteins [120]. Therefore, it might not represent a tissue-specific ECM environment for the ICO. Liver ECM extracts are a favorable alternative, since they are tissue-specific and could be of clinical relevance. The use of liver ECM extracts could unlock the full clinical potential of the cholangiocyte organoids.

## 6. Finding a Suitable Source of Liver ECM

In theory, the decellularization procedure removes all cellular components (including immunogenic proteins and DNA) from liver tissue. ECM components are also highly preserved between species, allowing for the use of animal liver-derived extracts for clinical applications in humans. However, retention of species-specific differences in liver ECM extracts have previously been reported by Loneker et al. [110]. These differences can partially be explained by biological variances between species, but also depend on tissue processing (e.g., decellularization and enzymatic digestion). Detergents can, for example, have detrimental effects on ECM components. We previously showed that triton-X-100 + Sodium dodecyl sulfate removes more collagen and sGAG from porcine liver ECM than when only triton-X-100 was used for decellularization [94]. Similar effects have also been reported by others who compared different treatment methods [100,121,122,123]. Decellularization of human livers required more detergent and longer exposure times compared to decellularization of similar sized porcine livers [94]. Subsequently, this could create larger differences between human and porcine livers. Therefore, it is important to consider the source of liver tissue.

Decellularized human livers are a promising allogeneic source of scaffolds for tissue engineering applications and for preparation of liver ECM extracts. However, healthy livers are relatively scarce. Human research livers (donor livers deemed unsuitable for transplantation) were used in our previous studies. Further improvements in the field of organ preservation and development of ex vivo repair strategies could in the future allow for the safe transplantation of these research livers [12]. Thereby, the number of available livers for research purposes will likely further diminish. Cadaveric livers could be an alternative source of healthy human livers. One of the disadvantages of the use of human livers is the relative old age and subsequent age-related changes of the ECM [124,125,126]. With increased age comes increased stiffness and decreased elasticity due to scar tissue formation and/or non-enzymatic crosslinking of the ECM [127]. The latter is caused by the age-related accumulation of advanced glycation end-products (AGE) attached to the ECM. This accumulation of AGE is influenced by different factors, such as dietary habits [128], and can increase the aforementioned cross-links. These changes in ECM can prevent enzymatic digestion of the ECM [113,125,129,130]. Subsequently, this can also lead to relatively large differences between human livers [124].

**Figure 3:**
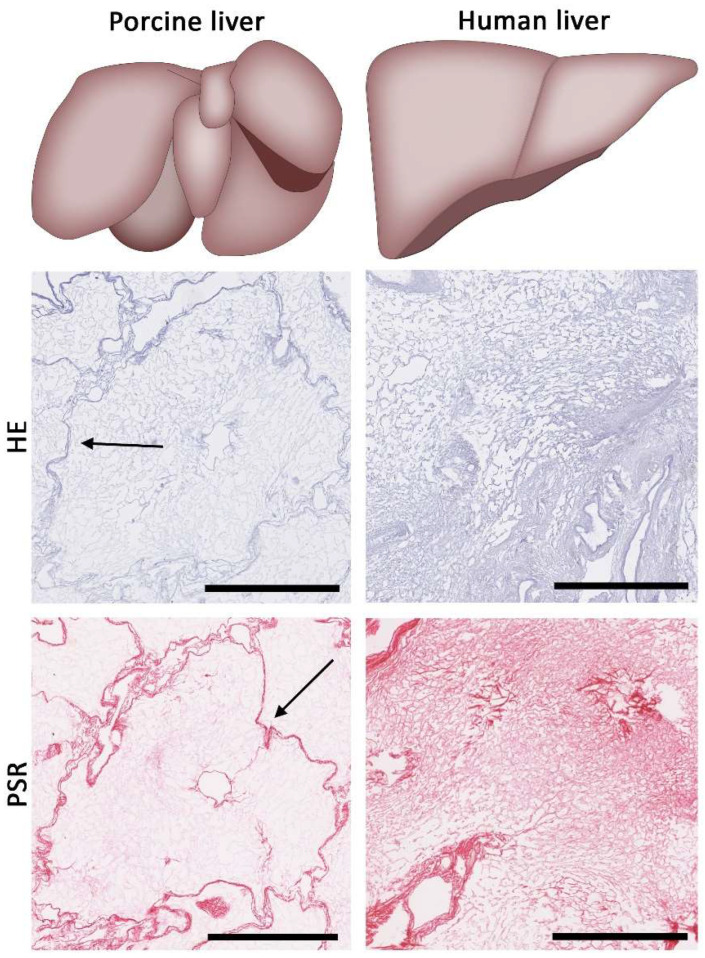
Porcine livers differ from human livers from an anatomical point of view. Porcine livers have multiple lobes, but also contain septa (indicated by black arrows). These septa are not visible in human livers. The scale bars represent 500 µm.

Animal livers of similar age can be obtained with relative ease in a standardized manner, thereby limiting the effect of age-related biological variances of the ECM. Small animal livers (e.g., mice, rat or ferret) are typically well suited for small-scale recellularization experiments. However, generating significant amounts of ECM extracts would require sacrificing many animals. Porcine livers, on the other hand, are comparable in size and weight to human livers. They are therefore promising alternatives for creating tissue-engineering scaffolds, but also yield more liver ECM extracts per liver. The anatomy of porcine livers is not similar to the anatomy of human livers. Porcine livers have 2–7 lobes, depending on breed [131]. Furthermore, the hepatic lobules of the porcine livers are separated by a clearly visible septa made of collagen-rich tissue (see Figure 3). These septa resemble fibrotic human livers and are not present in human livers under normal healthy circumstances [72,131]. The presence of these collagen-rich septa could shift the relative distribution of ECM components present in the liver ECM extracts and this could influence the behavior of primary human hepatocytes or stellate cells [124].

### Biosafety Concerns of Using Decellularized Liver Tissue

The use of decellularized tissues in clinical settings is associated with biosafety concerns. Inadequate processing of decellularized tissue can have detrimental effects on the scaffold and cause adverse clinical outcomes, such as rapid degradation, loss of mechanical integrity and/or inadequate tissue remodeling [132,133,134]. The presence of cellular remnants, especially xenogeneic components such as α-gal (galactose-α-1,3-galactose) or MHC proteins [135,136], can influence the host response to decellularized ECM or solubilized ECM (see [137,138] for comprehensive reviews on host response to animal-derived decellularized ECM).

Transmission of zoonotic diseases is another concern of using animal tissue-derived materials for clinical applications. Porcine endogenous retrovirus (PERV) is an example of a virus which could be transmitted [139]. Certain PERV subtypes can infect oversimplified human cell cultures in vitro, but so far, there are no reports of humans which have been infected with PERV after long-term exposure to pigs/porcine meat (e.g., farmers or butchers) or patient who received porcine corneas or islets of Langerhans [139,140]. Moreover, others have shown complete removal of detectable PERV provirus after complete decellularization of porcine tissue [140,141]. Of note, allogeneic liver transplantation is not without risk of transmitting human viruses [1]. Therefore, it is paramount that the decellularized livers and subsequent extracts are screened for contaminants, immunogenic components and (pro)viruses in order to mitigate these biosafety concerns.

## 7. Unlocking the Future Clinical Potential of Cholangiocyte Organoids

Tissue-specific ECM-based hydrogels are attractive culture substrates which can potentially unlock the full clinical potential of cholangiocyte organoids. This potential is not limited to use of the organoid-derived cells in cell therapy in ex vivo organ repair strategies. Patient-derived cholangiocyte organoids can retain patient characteristics and organoids cultures have been established for various hepatobiliary diseases, including alpha1antitrypsin deficiency [21], cystic fibrosis [27], Alagille syndrome [21], primary sclerosing cholangitis [142] and primary liver cancer [143,144]. Therefore, (personalized) in vitro disease models are an obvious application of the organoids (a comprehensive review on the use of hepatobiliary organoids for disease modeling is published by Nucifero and colleagues [145]). Cell–cell and cell–matrix interactions play important role during the development of hepatobiliary diseases, as the onset is often associated with significant changes in the ECM [144]. Decellularized liver tissue and liver ECM extracts can play important roles in creating these disease models. Moreover, the biological variances could also be embraced as a means to study the effect of different environments (e.g., relatively young ECM versus relatively old ECM [125,127]) on the behavior of cells or on the development of certain diseases [144]. Ultimately, patient-derived organoids cultured in tissue-specific matrices could lead to improved treatment strategies for hepatobiliary diseases.

### 7.1. Tissue Engineering the Biliary Tree

Tissue-engineered functional liver constructs have the potential of bridging the gap between the demand and supply of donor livers of adequate livers [146,147,148]. Creating liver constructs in vitro requires scaffolds that are capable of performing similar roles as the native liver ECM. Current production techniques for alternative ECM or synthetic supporting structures, such as 3D bio-printing, can recreate small constructs mimicking the liver architecture with high fidelity, but producing clinically relevant sized scaffolds is still challenging [149,150]. Use of the native liver ECM is an attractive alternative to de novo creation of scaffolds with synthetic materials [97,147,148]. In recent years, recellularization with primary hepatocytes or hepatocytes derived from various stem cell sources have been investigated in effort to restore functionality of the hepatocyte compartment [71,151,152,153]. Simultaneously, a lot of effort has been invested in repopulation of the vasculature network [95,148]. However, repopulation of the biliary compartment has not yet been extensively studied, even though biliary epithelium is essential for proper functioning of the liver [9,154].

**Figure 4:**
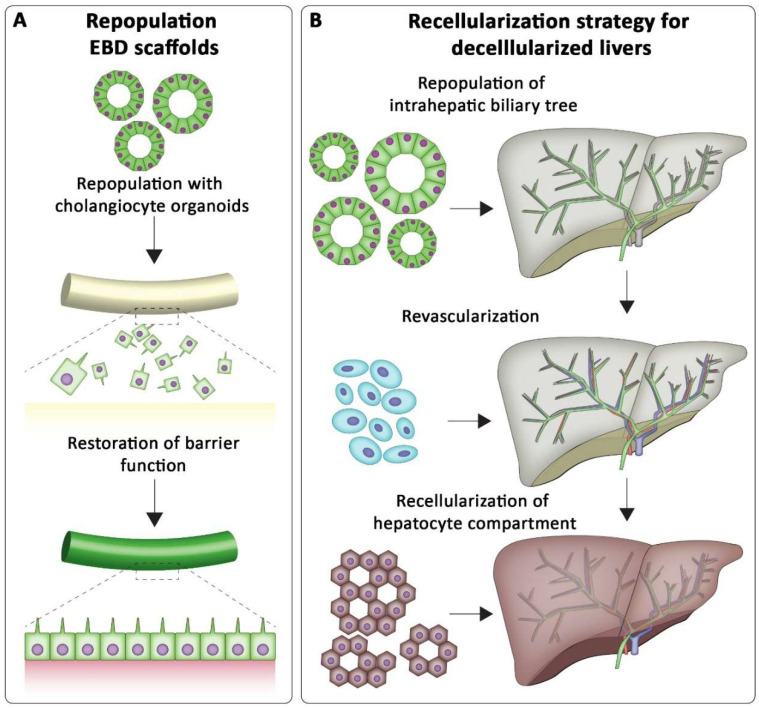
Potential future tissue engineering applications of human cholangiocyte organoids. (**A**) Cholangiocyte organoids can also be used to repopulate extrahepatic bile duct (EBD) scaffolds for ductal tissue engineering purposes. Subsequently, these engineered ductal scaffolds can be used to replace damaged tissue. (**B**) An example of a strategy for recellularization of decellularized liver ECM. Cholangiocyte organoids can be used to repopulate the entire biliary tree. Endothelial cells can recellularize the vasculature of the liver and ICO differentiated towards hepatocyte-like cells can be used to repopulate the hepatocyte compartment. These different types of cells could restore functionality of the liver.

Cholangiocyte organoid-derived cells are a promising source of cells for repopulation of the biliary tree. We recently showed that cells derived from ECO and BCO were capable or repopulating small discs (Ø3 mm) of decellularized extrahepatic bile duct (EBD) tissue [30,155]. They self-organized into polarized monolayers resembling biliary epithelium (Figure 4). Moreover, the repopulated scaffold showed increased trans epithelial electrical resistance and cholangiocyte specific ion-channel activity could be measured. This could be translated into transplantable EBD constructs, but the organoids could also be used to repopulate the biliary tree of decellularized liver ECM (Figure 4) [25,156]. However, also here the use of BME as a culture substrate hampers the clinical applications of these lab-grown biliary structures. Clinical grade alternatives are required for the in vitro expansion of organoids, before repopulated biliary trees can be used in vivo.

Together with more liver-specific cell types, such as hepatic stellate cells and Kupffer cells, a transplantable tissue-engineered liver construct can be made. Decellularized liver tissue can be applied in different stages and in different forms for creating tissue-engineered liver constructs in vitro. Solubilized liver ECM hydrogel can, for example, be used for the clinically relevant expansion of cholangiocyte organoids in vitro, while decellularized livers can be used as a bioactive, biodegradable and inductive scaffold for liver tissue engineering purposes in vitro and in vivo.

### 7.2. Summary

Mouse tumor-derived BMEs are commercially available and easy-to-use formulations which allow for organoid growth and expansion. However, these one-size-fits-all BME formulations limit the direct clinical application of cholangiocyte organoids for cell therapy or tissue engineering applications. In addition, BME lacks essential tissue-specific ECM components and is known to keep cells in a high proliferative undifferentiated state. There is a clear need for a tissue-specific alternative which can be produced according to GMP guidelines. This alternative must also allow for the culture and large-scale expansion of these organoids. Hydrogels derived from healthy decellularized and solubilized liver ECM are promising alternative culture substrates for the large-scale expansion of cholangiocyte organoids and could unlock the enormous clinical potential of the organoids.
